# How tightly are production and comprehension interwoven?

**DOI:** 10.3389/fpsyg.2013.00238

**Published:** 2013-04-26

**Authors:** Martin J. Pickering, Simon Garrod

**Affiliations:** ^1^Department of Psychology, University of EdinburghEdinburgh, UK; ^2^Institute of Neuroscience and Psychology, University of GlasgowGlasgow, UK

MacDonald's (2013) production-distribution-comprehension (PDC) account provides a valuable unification of three aspects of language use. It identifies some of the principles involved in production and shows how they affect both distributions of linguistic forms and the process of comprehension. Our commentary presents a complementary perspective on the relationship between production and comprehension.

On the basis of extensive evidence, the PDC account proposes that speakers tend to utter easy-to-produce words early, reuse recently used structures, and avoid producing utterances in which elements interfere with each other. These principles produce distributions of utterances, and comprehenders develop processing mechanisms that are sensitive to these distributions. Thus, they find it easier to interpret utterances that accord with these principles than utterances that do not accord with them, and use these principles to guide ambiguity resolution. In other words, comprehenders are fundamentally affected by their experience of language and hence by its statistics.

The relationship between production and comprehension is mediated by distribution. There is no direct (“on-line”) effect of production on comprehension. This contrasts with our *integrated* account of production and comprehension, in which people use production processes to guide comprehension (and in fact use comprehension processes to guide production). Pickering and Garrod (in press) argued that production and comprehension are not isolated from each other, but that instances of comprehension can involve production processes, rather than “feedback” within the comprehension system (and that instances of production can involve comprehension processes). We now propose that this interweaving in fact provides the basis for the relationships between production and comprehension identified by MacDonald.

Pickering and Garrod (in press) argued that addressees construct predictions of what they are about to hear before they hear it (see also Pickering and Garrod, 2007). Such predictions make use of *forward models*, as assumed in many accounts of action and action perception (see Wolpert, 1997). For example, if I decide to move my hand to a particular location, I combine my intention, my hand's position in relation to the environment, and my experience of the outcome of previous similar intentions to construct a representation of (the percept of) my predicted hand movement. For example, I might predict that a particular intention will cause my hand to move 500 mm from my body and 30° left of my midline, in 300 ms time. Importantly, the forward model output (i.e., prediction) will be ready in (say) 100 ms, before the movement. In fact, I need to construct this model in order to learn to control the movement fluently (Wolpert et al., 2001). I can then compare the prediction with the actual movement when it occurs.

When I see you starting to move your hand, I construct a prediction of where your hand will end up, again before you move your hand, and then compare this with your actual movement. Pickering and Garrod (in press) proposed that perceivers can do this by determining what they would do if it were their hand (using “prediction-by-simulation”). In other words, I covertly imitate your movements to determine the intention behind your movement and use that intention to predict (my percept of) your movement. The mapping from intention to prediction involves the same forward model as when I move my own hand, which constitutes part of the action system.

Pickering and Garrod (in press) noted that language comprehension is a form of action perception. They therefore proposed that comprehenders covertly imitate what they hear to determine the production command (roughly, the speaker's intended message), and then use that command to predict (their percept of) the unfolding utterance. This mapping involves a forward model that forms part of the production system. Importantly, comprehenders can predict linguistic representations concerned with meaning, grammar, or sound. For example, the comprehender hears the start of the speaker's utterance and uses covert imitation to predict aspects of the upcoming utterance, for example that the upcoming word will refer to something edible (Altmann and Kamide, 1999) or will begin with a vowel (DeLong et al., 2005).

One consequence of this framework is that ease of comprehension is affected by ease of production. For example, people utter easy-to-produce words early, so they predict that they will do so. They therefore also predict that their interlocutor will utter easy-to-produce words early. Similarly, MacDonald (2013) pointed out that this easy-first principle discourages production of utterances such as *John will say that his cousins left tomorrow* (as *tomorrow* is short), and hence that such utterances are rare. We argue that comprehenders hear the sentence up to *left*, and then use their production system to predict that their interlocutor will produce a local modifier. This prediction involves the rapid construction of a forward production model, which therefore tends to be ready before the speaker utters *tomorrow*. At this point, the comprehender can compare the predicted and actual utterance percepts (using what we term other-monitoring). In this case, the important component of the prediction is likely to be syntactic (relating to the constituent structure of local modification) and semantic (relating to prior temporal modification).

Our framework applies similarly to *plan reuse* and *reduce interference*. People tend to repeat syntactic structure, so they predict that they will do so, and so they also predict that their interlocutor will do so. Finally, people disfavor utterances involving proximate interfering words, so they predict they will not produce such utterances, and so they also predict that their interlocutor will not do so either. If they hear a sentence with a different syntactic structure or interfering words in close succession, they will tend to experience more difficulty than otherwise; and if an utterance is ambiguous, they will tend to assume that it involved syntactic repetition or that it did not involve interference.

Importantly, prediction-by-simulation involves determining the speaker's intention, and this involves combining the speaker's utterance so far with the non-linguistic context. Just as I use my knowledge about our relationship to predict whether your arm movement is prelude to a handshake or a punch, so I use non-linguistic context to determine whether you are likely to complete *I'm thirsty, could you get me a…* with *coffee* or *beer*. We propose that people can use another route to prediction (prediction-by-association), based on usage statistics, and which is no different for complex non-linguistic experiences that need not involve action perception (e.g., perception of inanimate objects). It appears that PDC makes use of such statistics, though it makes no claim about prediction. We argue that prediction-by-simulation is much more versatile because it incorporates non-linguistic context to derive intentions. For example, it can straightforwardly explain the immediate detection of the anomaly of a man's voice saying “I'm pregnant” (Van Berkum et al., 2008). A statistical account of such anomaly detection is far from apparent.

The PDC account appears to assume “autonomous transmission” in which production and comprehension are independent processes (Pickering and Garrod, 2004). However, autonomous transmission is difficult to reconcile with the fluency of dialog, in which interlocutors overlap their speech (thus producing and comprehending at the same time) and regularly complete each other's utterances. In (1) below [from Gregoromichelaki et al. (2011)] *B* begins to ask a question (1b), but *A*'s interruption (1c) completes the question and answers it.

1a—–A: I'm afraid I burnt the kitchen ceiling
1b—–B: But have you
1c—–A: burned myself? Fortunately not.

The fluency of such joint contributions suggests that comprehension and production processes are directly linked in dialog. This is difficult to explain in an autonomous transmission framework in which *A* has first to comprehend the utterance (1b) and then independently produce the continuation (1c) from scratch (see Pickering and Garrod, in press). However, we believe that the principles of the PDC account are in fact compatible with our proposal, in which production and comprehension are tightly interwoven.

We therefore applaud MacDonald's (2013) insistence on the relationship between production and comprehension (as well as distribution). But the importance of prediction-by-simulation and the tight relationship between contributions to dialog suggests that the relationship between production and comprehension is more direct than assumed by the PDC account.
